# Binding Constants of Substituted Benzoic Acids with Bovine Serum Albumin

**DOI:** 10.3390/ph13020030

**Published:** 2020-02-20

**Authors:** Diliara Khaibrakhmanova, Alena Nikiforova, Igor Sedov

**Affiliations:** Chemical Institute, Kremlevskaya 18, Kazan Federal University, 420008 Kazan, Russia; dilusechka@gmail.com (D.K.); alnikiforova22@gmail.com (A.N.)

**Keywords:** albumin, binding constant, benzoic acids, spectrofluorimetry, Hammett constants, QSAR

## Abstract

Experimental data on the affinity of various substances to albumin are essential for the development of empirical models to predict plasma binding of drug candidates. Binding of 24 substituted benzoic acid anions to bovine serum albumin was studied using spectrofluorimetric titration. The equilibrium constants of binding at 298 K were determined according to 1:1 complex formation model. The relationships between the ligand structure and albumin affinity are analyzed. The binding constant values for m- and p-monosubstituted acids show a good correlation with the Hammett constants of substituents. Two- and three-parameter quantitative structure–activity relationship (QSAR) models with theoretical molecular descriptors are able to satisfactorily describe the obtained values for the whole set of acids. It is shown that the electron-density distribution in the aromatic ring exerts crucial influence on the albumin affinity.

## 1. Introduction

Experimental in vivo study of plasma protein binding is an important step in development of novel drug substances. The strength of binding affects the distribution of a drug in the body along with its pharmacodynamic parameters, biological activity and toxicity [1]. The most abundant protein in blood plasma is serum albumin. It can be extracted from plasma and used to study binding processes in vitro. In such experiments, the strength of binding is characterized quantitatively using thermodynamic binding constants. The measured values of these constants can be used to estimate the fractions of plasma-bound and free drug in the blood, which helps to reduce the number of costly in vivo experiments [2].

**Figure 1:**
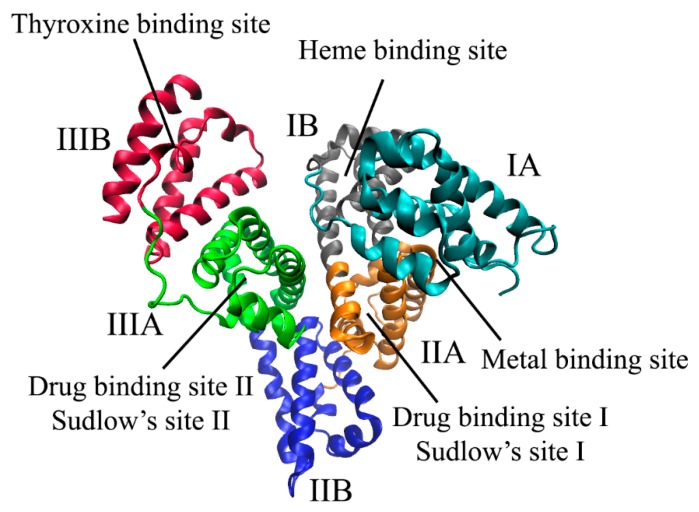
Positions of binding sites in a bovine serum albumin molecule.

However, accurate determination of the binding constant values is complicated by many factors. In particular, the albumin molecule contains several binding sites with different affinity to different species [3] (Figure 1). Furthermore, albumin partially dimerizes in solution, and each binding site of the dimeric form may change its affinity to the ligands [4]. For bovine serum albumin, the dimeric form is predominant at concentrations above 5 μM (*K* = 10 ± 2 μM [5]). 

Moreover, none of the commonly used experimental techniques allows accurate determination of separate binding constants for each site. The experimental data are always analyzed using some simplified binding models, and the obtained binding constant values are in fact some complicated functions of the site-specific constants. Thus, the results are heavily dependent on the method of measurement and data processing. The existing literature data for the same ligand from different authors often contradict each other and are unreliable (see below for the examples).

Even though the method-dependence of results is inevitable, the affinities of different compounds to albumin can be still compared if the experiments are conducted at similar conditions. In other words, a set of compounds should be studied using the same technique with albumin from the same source taken at the same concentration, and the data should be analyzed according to the same binding model. Only such uniformly obtained data can be used to develop quantitative structure–property relationships or other models for a priori estimation of affinity in virtual screening [1]. However, most of the extremely numerous works providing binding constant values are limited to a single ligand or a small number of ligands.

Serum albumin is rather nonselective and can bind ligands with different molecular size, shape and functional groups. In the present work, we limit our study with a series of substituted benzoic acids. Variation of positions and types of substituents in the aromatic ring is an easy way to create a set of compounds with different electronic structure and hydrogen-bonding properties, which was widely used in the studies of organic reactivity [6]. In addition, many derivatives of benzoic acid are biologically and pharmacologically active. Analysis of the influence of the nature and position of aromatic ring substituents on the toxicities and activities of these compounds led to the development of the first quantitative structure–activity relationships (QSARs) [7]. Thus, the substituted benzoic acids are interesting and convenient objects for accumulation of experimental albumin binding data and studying the links between the structure and affinity of ligands.

Several previous works were devoted to binding of substituted benzoic acids to albumin. Matsushita et al. [8] determined the binding constants of 14 p-substituted benzoic acids to human and bovine serum albumin using ultracentrifugation method. The results were analyzed using multiple linear regressions with the ligand properties such as molecular volume and dissociation constant p*K*_a_. It was concluded that large ligand size and strong acidity favor binding. Moriguchi studied binding of a series of 14 o-, m-, and p-substituted benzoic acids to bovine serum albumin using spectrophotometry [9]. The logarithms of the obtained binding constants values for m- and p-substituted acids correlated with pK_a_ values of the acids, showing stronger binding for stronger acids. However, o-substituted benzoic acids did not follow this correlation, which was attributed to the ortho-effect. Authors suggested that these results prove that binding is due to the interactions between carboxylic group and cationic groups of amino acid residues in albumin. Two recent works made by Hao Zhang et al. are devoted to spectrofluorimetric determination of binding constants of 111 substituted benzoic acids to human serum albumin [10] and 114 substituted benzoic acids to bovine serum albumin [11] and development of the structure–affinity relationships. Despite such a large number of acids, only combinations of three different substituents (–OH, –OCH_3_, and –CH_3_) in aromatic ring were considered. Correlations of the obtained values with any single ligand parameter including pK_a_ were quite poor. Some observations on the influence of substituents were made, in particular, it was concluded that o-hydroxylation increases the affinity, while p-hydroxylation decreases it.

In the present work, binding of 24 substituted (mostly monosubstituted) benzoic acids to bovine serum albumin at 298 K is studied. Correlations of the obtained binding constants with various ligand properties are considered, and the possibility of their a priori estimation using QSAR approach is analyzed.

## 2. Methodology

### 2.1. Binding Centers of Albumin

**Figure 2:**
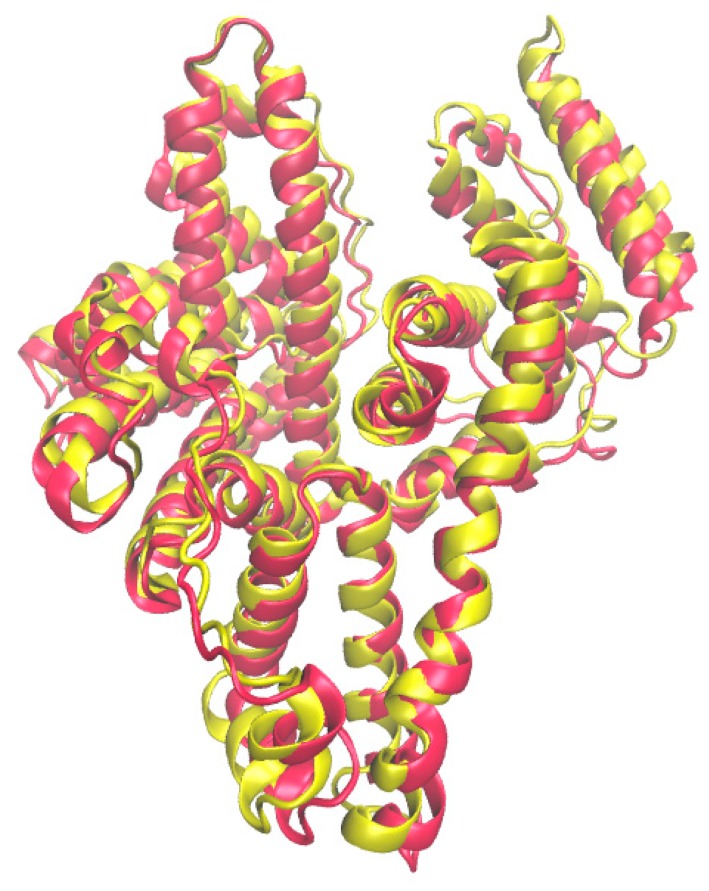
Comparison of the spatial structures of bovine (red) and human (yellow) serum albumin.

Albumins from the plasma of different mammals are globular proteins consisting of three homologous domains (I, II, III). Each of them contains two subdomains, A and B [12]. Human serum albumin (HSA) and bovine serum albumin (BSA) (Figure 2) attract the most attention from researchers, however, the processes of binding to albumin from the blood plasma of other animals, such as rabbit, dog and horse, have also been studied [13,14,15]. All these albumins have minor differences in structure.

Most of the drug molecules are thought to bind with two high affinity binding sites in albumin subdomains IIA and IIIA [16], called Sudlow I and Sudlow II, respectively. According to the numerous publications, Sudlow I is preferred by bulky heterocyclic molecules and Sudlow II is preferred by aromatic carboxylates [17]. Since the primary amino acid sequence of bovine serum albumin is 75.6% identical to that of human serum albumin [18], their binding centers are also similar. The amino acid residues in Sudlow II are the same, however, for Sudlow I only 57% identity was shown [18]. Bovine serum albumin has arginine residues in 195 and 199 positions instead of lysine in Sudlow I site. This change may cause some difference in binding properties.

### 2.2. Spectrofluorimetric Method and Its Pitfalls

Spectrofluorimetry is a common method to study protein–ligand interactions. Tryptophan, tyrosine and phenylalanine can contribute to the intrinsic fluorescence of proteins. When protein–ligand interactions occur, fluorescence quenching is usually observed due to the changes in the microenvironment of amino acid residues. This phenomenon can be used to determine the binding constant values. In such studies, the excitation wavelength 295 nm is often used. At this wavelength, fluorescence is predominantly caused by tryptophan residues. Bovine serum albumin has two tryptophans: Trp-212 located in Sudlow I binding site and Trp-134 in subdomain IA while human serum albumin has only one Trp-214 in Sudlow I [19]. However, binding to each of the sites can quench the fluorescence. The quenching is monitored by following the fluorescence intensity at the wavelengths near the emission maximum (340–355 nm).

In fluorescence experiments, the concentration of ligand is most commonly varied, and the signal change is analyzed. There is no way to obtain the data on binding with separate sites of albumin from such experiments. A very simple model of 1:1 binding is usually used. Thus, we should avoid a large excess of ligand, especially in the case of strong binding. The most common way to calculate the binding constant is using the Stern–Volmer equation relating the fluorescence of protein in the absence and in the presence of ligand (*F*_0_ and *F*, respectively) to the total concentration of added ligand ${\left[L\right]}_{\Sigma }$ and the binding constant *K*:

(1) $$\frac{F_{0}}{F}=K{\left[L\right]}_{\Sigma }+1,$$

Derivation of this equation relies on several assumptions. As mentioned above, the first one is 1:1 complex stoichiometry. Second, there should be only one type of fluorophore in protein. This is not true for bovine serum albumin, but always neglected. Third, bound ligand should quench fluorescence intensity down to zero. This is not true for all ligands. Fourth, the equilibrium concentration of the bound ligand should be negligible. This is a problem only in the case of strong binding and can be overcome by correcting the obtained values, e.g., using numerical simulations of Stern–Volmer plots for various large constant values.

If all these assumptions are correct, then for the binding process

Alb + L = AlbL

with the binding constant

(2) $$K=\frac{\left[\mathrm{AlbL}\right]}{\left[\mathrm{Alb}][L\right]},$$

the total albumin concentration in solution is given by

(3) $${\left[\mathrm{Alb}\right]}_{\Sigma }=[\mathrm{Alb}]+K[\mathrm{Alb}]{\left[L\right]}_{0},$$

thus

(4) $$\frac{F_{0}}{F}=\frac{{\left[\mathrm{Alb}\right]}_{\Sigma }}{\left[\mathrm{Alb}\right]}=K{\left[L\right]}_{\Sigma }+1.$$

The use of the methods based on fluorescence quenching and particularly the Stern–Volmer equation was an object of severe criticism [20,21]. In addition to the above-mentioned pitfalls, a common mistake leading to unreliable constant values is the neglect of the inner filter effect caused by significant absorbance of solution at the excitation or emission wavelength. Moreover, fluorescence can be quenched by a dynamic collisional quenching mechanism not related to the formation of the protein–ligand complex. The shift of the fluorescence peak upon binding can also lead to incorrect results. In the present work, we avoid most of the known pitfalls. We also believe that the rest of the possible systematic errors and the limitations of the simplified binding model will affect the whole series of benzoic acids in the same manner so we at least can compare the relative strength of their binding with albumin.

## 3. Experimental

### 3.1. Chemicals and Instruments

All the chemicals were commercial products and were used without further purification. Fluorescence measurements were performed using Cary Eclipse fluorescence spectrophotometer (Agilent Technologies, Santa Clara, CA, USA).

### 3.2. Spectrofluorimetric Titration Procedure

In a typical experiment, a fresh solution of bovine serum albumin (PanEko, Moscow, Russia, defatted, purity > 98%) with a concentration of about 1 µM in phosphate buffer (pH 7.4, 0.05 M) was prepared. Ligands (acids) were dissolved in the same buffer and then diluted to concentrations in the range of 20–50 µM. According to the p*K*_a_ values of the studied acids, all of them exist almost entirely in ionized form at such pH value. All solutions were thoroughly degassed by stirring under vacuum. Next, 2.3 mL of protein solution was poured into a quartz spectrofluorometric cuvette and allowed to settle for 40 min to saturate the walls of the cell with adsorbed protein molecules. Then, the protein solution was replaced with a fresh one and titrated with 10 µL portions of ligand solution using an automated syringe pump equipped with a 250 μL Hamilton microsyringe with a long steel cannula. The endpoint of titration usually corresponded to ${\left[L\right]}_{\Sigma }:{\left[\mathrm{Alb}\right]}_{\Sigma }\approx$2:1. The cuvette holder had a built-in Peltier thermostat keeping temperature equal to 298 K and a magnetic stirrer. After addition of each portion of ligand solution, the fluorescence spectrum with the excitation wavelength λ_ex_ = 295 nm and emission wavelengths λ_em_ = 310–360 nm was recorded. Additional spectrofluorimetric measurements of absorbance of solutions before and after titration at λ_ex_ and λ_em_ have shown that the influence of the inner filter effect is negligible. The fluorescence intensities at the emission maximum, 347 nm, were taken for further calculations. The studied acids do not have a significant fluorescence signal at this wavelength (p-aminobenzoic acid was not included in the study because it does).

### 3.3. Evaluation of the Binding Constants

In order to use Stern–Volmer equation (Equation (4)), the fluorescence intensity should be measured for several solutions with the same concentration of protein but at different concentrations of ligand [21]. However, during titration the protein solution is being diluted at each step. Thus, the ratio of the fluorescence intensity to the current total concentration of albumin should be used instead. The equation used to calculate the binding constant has the following form:

(5) $$\frac{\varepsilon_{0}}{\varepsilon }=K{\left[L\right]}_{\Sigma }+1,$$

where $\varepsilon_{0}=\frac{F_{0}}{{\left[\mathrm{Alb}\right]}_{\Sigma 0}}$, $\varepsilon =\frac{F}{{\left[\mathrm{Alb}\right]}_{\Sigma }}$, ${\left[Alb\right]}_{\Sigma 0}$ is the initial total albumin concentration and ${\left[\mathrm{Alb}\right]}_{\Sigma }$ is the total albumin concentration after dilution with ligand solution. In a control titration with buffer solution containing no ligand, the values of ε remained equal to $\varepsilon_{0}$.

The plots of $\frac{\varepsilon_{0}}{\varepsilon }$ against ${\left[L\right]}_{\Sigma }$ allow to determine the value of the binding constant, which is equal to the slope of the linear correlation. These values were averaged over 4–5 titrations with freshly prepared albumin and ligand solutions.

The constants of binding of the studied compounds to BSA appear to be not very large, so that no more than 10% of ligand is bound in solution. Nevertheless, we have written a program for numerical simulations of Stern–Volmer plots for titration with a given binding constant and concentrations of BSA and ligand solutions. (see the Supplementary Materials for the source code and example of simulation of 4-nitrobenzoic acid titration in Wolfram Mathematica nb format). It helps to find the value of *K* providing the best fit of the simulated curve to the experimental data. The difference between the apparent and corrected values of *K* was not great: for the largest apparent constant 7.6 × 10^4^ (4-nitrobenzoic acid) the corrected value equals 8.1 × 10^4^. Table 1 lists the final corrected values for all the studied systems. The standard state of the concentrations in the expression (Equation (2)) for the binding constants is 1 M.

## 4. Discussion

### 4.1. Literature Albumin Binding Data for the Studied Compounds

For some of the studied benzoic acids, the binding constants have been previously measured by different authors using different methods and reported in the literature [8,9,10,11,22,23,24]. Their results (for binding with both BSA and HSA) are listed in Table 2. The pH of buffer solutions used in these studies varied from 7.0 to 8.50. It can be seen that the values from different works strongly disagree with each other. As discussed above, the binding constant values are heavily method dependent. There is no point in direct comparison with our results, even if there are some occasional agreements. The same trend of decrease of *K* values in a row for 2-hydroxy > 3-hydroxy > 4-hydroxybenzoic acid observed in the present work and in two previous studies [9,11] provides some evidence of the consistency of results, but consideration of the structure–affinity relationships should be limited to the data obtained using exactly the same experimental procedure.

### 4.2. Correlation of the Binding Constants with Hammett Substituent Constants

We have not found a good correlation of the binding constants for the whole set of 24 substituted benzoic acid with any single physicochemical parameter of ligand. However, the values of log*K* for a subset of m- and p-monosubstituted acids correlate with the Hammett constants σ of the substituents:

log*K* = 0.79σ + 4.34 RMS = 0.13, *n* = 15, *r*^2^ = 0.8257. (6)

**Figure 3:**
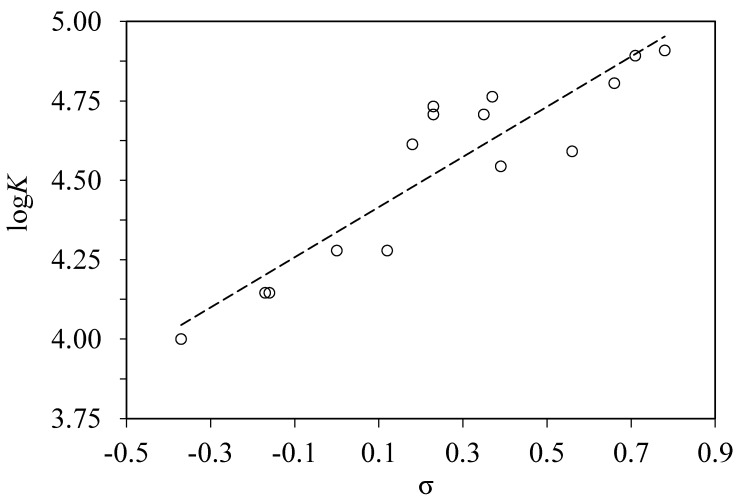
Correlation of the logarithms of binding constants log*K* of m- and p-monosubstituted benzoic acids to BSA with the Hammett constants σ [25] of the substituents.

This correlation is shown in Figure 3. The interactions involving o-substituted benzoic acids is heavily influenced by the steric effects of substituents. Their behavior in any physical or chemical process cannot be interpreted in terms of Hammett constants, which are not defined for o-substituents as well as for disubstituted derivatives [25].

The Hammett constants describe the electron donating or withdrawing ability of a substituent in comparison with a hydrogen atom. Equilibrium and rate constants of various chemical processes involving substituted benzoic acids derivatives and other aromatic compounds have been shown to correlate with these constants [26,27]. The applications of this approach to protein–ligand binding are quite limited. Previously, the binding constants of p-substituted benzamidines to trypsin were shown to have some correlation with the Hammett constants of substituents [28]. Enthalpies and entropies of binding of p-substituted acetanilides to BSA have shown a similar correlation [29].

The noncovalent interactions of m- and p-substituted benzoic acids with BSA molecule seem to follow the generic pattern observed for interactions with small organic molecules.

### 4.3. Correlations with Other Experimentally Derived Ligand Properties

**Figure 4:**
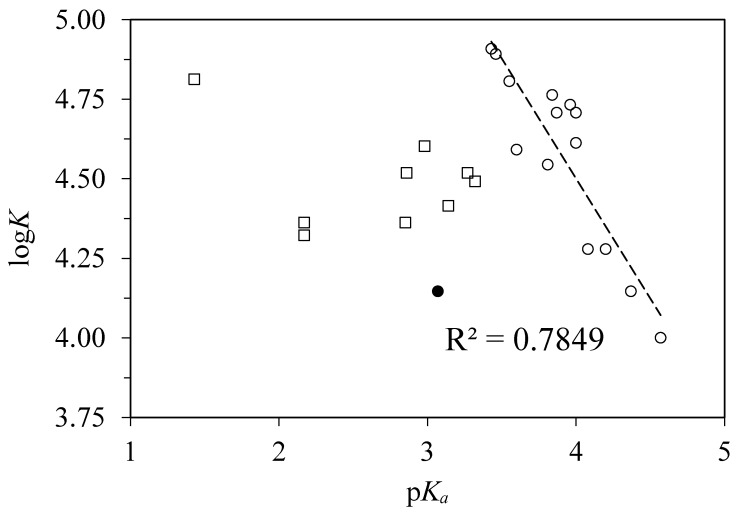
Plot of the logarithms of binding constants log*K* of substituted benzoic acids to BSA against their p*K*a values [31]. Empty circles represent m- and p-substituted acids, filled circle represents 3-aminobenzoic acid, empty squares represent o- and o,p-substituted acids.

Since the Hammett constants of m- and p- substituted benzoic acids are derived from the logarithms of their dissociation constants p*K*_a_ in water [25], it is not surprising that the binding constants to BSA also correlate with the p*K*_a_ values (see Figure 4). This correlation does not hold for o-substituted acids. Another exception is 3-aminobenzoic acid for which the Hammett constant was derived using the dissociation constant in alcohol–water mixture different from that in pure water [30].

**Figure 5:**
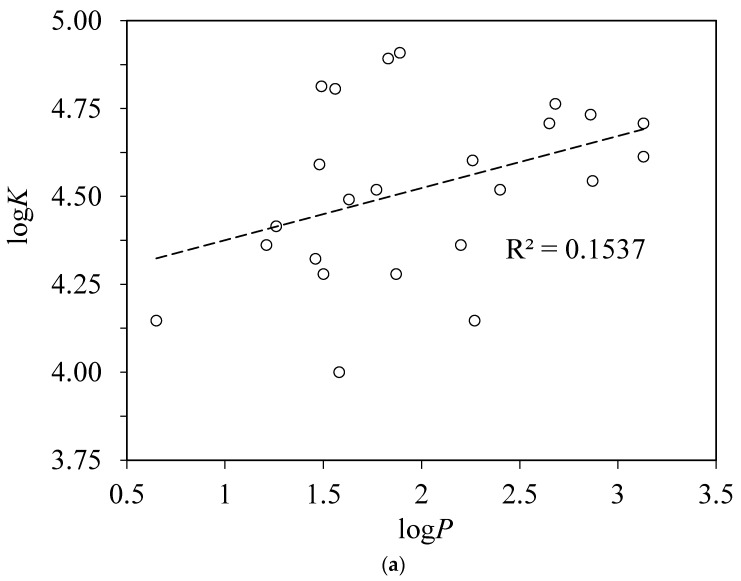

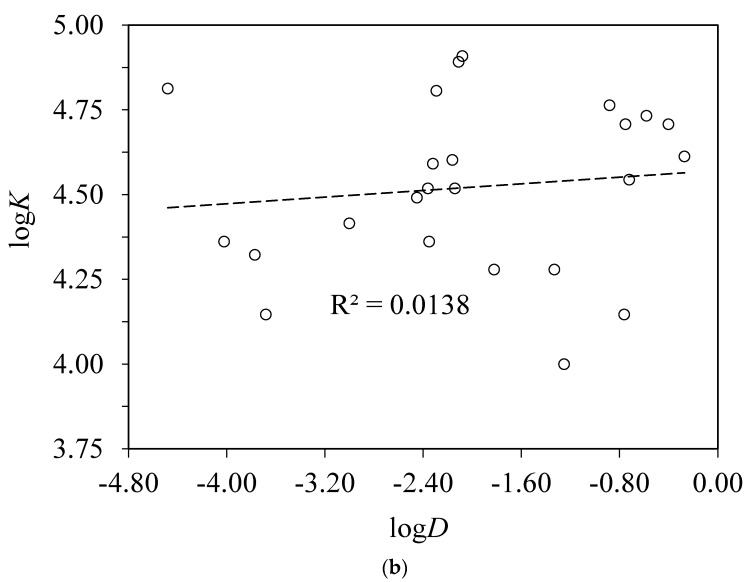
Plots of the logarithms of binding constants log*K* of substituted benzoic acids to BSA against their octanol–water partition coefficients log*P* [34] (**a**) and log*D* (**b**).

There were attempts to compare or correlate the albumin binding constants of different compounds with their octanol–water partition coefficients log*P* [1,2,24,32,33]. The quality of such correlations was not good, and it was not good in our case as well. For acidic ligands, it may be more correct to consider the distribution coefficient log*D* of both ionized and nonionized forms between octanol and water, which is related to the distribution coefficient of nonionized form log*P* through equation log*D* = log*P* − log(1 + 10^pH–p*K*a^). Plots of log*K* against log*P* and log*D* are shown in Figure 5.

**Figure 6:**
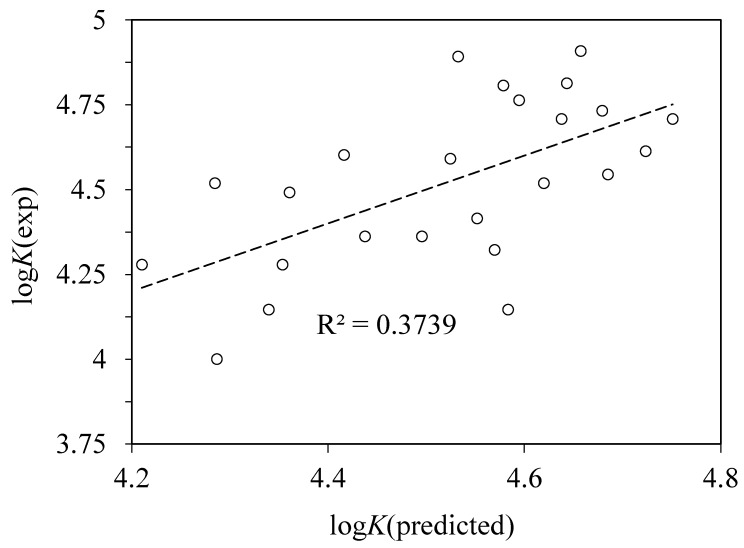
Plot of the experimental logarithms of binding constants log*K* against those predicted using a multiple linear regression with Abraham molecular descriptors E, S, A, B, and V [35].

In the works [2,32], the affinities of a large series of compounds, predominantly drugs, to immobilized HSA determined using HPLC method were correlated with a set of Abraham molecular descriptors of these compounds (*E*, *S*, *A*, *B*, *V*) [35], which show a good predictive ability in describing partition of many different compounds between different solvents and biological fluids. We performed a linear regression with these five descriptors, which led to a low-quality correlation with *r*^2^ = 0.374 (Figure 6). One may argue that the acidity descriptor A is irrelevant when we consider binding of anionic forms of acids which have lost their acidic proton. The analysis performed in the work [32] showed that only the parameters S, B, and V are significant for description of binding of a large set of structurally diverse molecules to an immobilized HSA stationary phase in HPLC experiments. Regression of our results with these three descriptors led to a lower coefficient of correlation *r*^2^ = 0.348. Moreover, we have considered all the possible linear regressions involving from one to five Abraham descriptors, and all of them had even lower values of *r*^2^. Adding log*P* and p*K*_a_ parameters to these regressions does not significantly improve the correlation quality. For the whole set of 24 ligands, the best possible correlation with one to seven of these parameters has *r*^2^ = 0.407.

### 4.4. QSAR Modeling of Albumin Binding

An alternative approach is based on QSAR modeling in which regressions with theoretically calculated descriptors are used. Previously, Colmenarejo et al. [36] developed a nonlinear QSAR model with six descriptors to describe the binding affinities of a set of 95 structurally diverse compounds to an immobilized HSA stationary phase. The *r*^2^ value for the best model was 0.83. Xue et al. [37] examined linear QSAR models for the same set of compounds and found a seven-parameter model with *r*^2^ = 0.86.

We considered linear QSAR models with two sets of descriptors. One of them was generated using PaDEL-Descriptor software [38] and included 1410 2D, 1D and 0D descriptors. Another set was generated by eDragon software [39] and included more than 1600 3D, 2D, 1D and 0D descriptors. The descriptors having the same value for all the ligands and strictly intercorrelated descriptors were eliminated. This left 874 descriptors from the first set and 1159 descriptors from the second set.

With the first set, we initially considered all the possible regressions with two different descriptors (381,501 combinations). The highest correlation coefficient achieved was *r*^2^ = 0.739:

log*K* = 3.501BCUTw-1l + 1.324MATS3c − 37.295. RMS = 0.124, *n* = 24, *r*^2^ = 0.739 (7)

**Figure 7:**
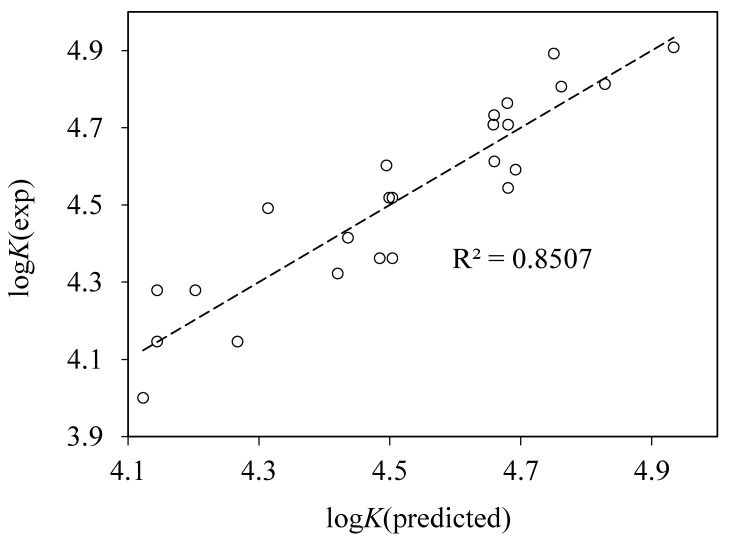
Plot of the experimental vs. predicted logarithms of binding constants log*K* obtained using the best three-parameter model (Equation (8)) with parameters from PaDEL descriptor set.

The first 40 top scoring correlations included BCUTw-1l descriptor. Next, all the possible regressions with three descriptors, one of which was always BCUTw-1l, were considered. The best one had *r*^2^ = 0.851 and is given by the following equation (Figure 7):

log*K* = 3.601BCUTw-1l + 0.0458MPC7 − 0.5258SP-4 − 37.706. RMS = 0.094, *n* = 24, *r*^2^ = 0.851 (8)

Additionally, a search for correlations with three different descriptors was performed using a genetic algorithm. No better correlations were found. The use of four descriptors leads to a further increase in the quality of correlations (up to *r*^2^ = 0.914 for the following correlation):

log*K* = 3.476BCUTw-1l − 4.748AATSC8c − 1.435GATS3c − 5.809 SpMin1_Bhs − 25.099. RMS = 0.071, *n* = 24, *r*^2^ = 0.914 (9)

The importance of the BCUTw-1l descriptor arises from its calculation algorithm. This descriptor is a weighted version of the Burden matrix [40] which takes into account the connectivity, charge and polarizability of atoms in a molecule [41]. It incorporates the information about the electron density in benzene ring. Despite BCUTw-1l for m- and p-monosubstituted benzoic acids being not strictly correlated with the Hammett constant of substituents, its values are in general lower in the presence of electron donating and higher in the presence of electron accepting groups. Some other applications of descriptors from BCUT family in description of protein–ligand interactions have been previously demonstrated [42,43,44].

**Figure 8:**
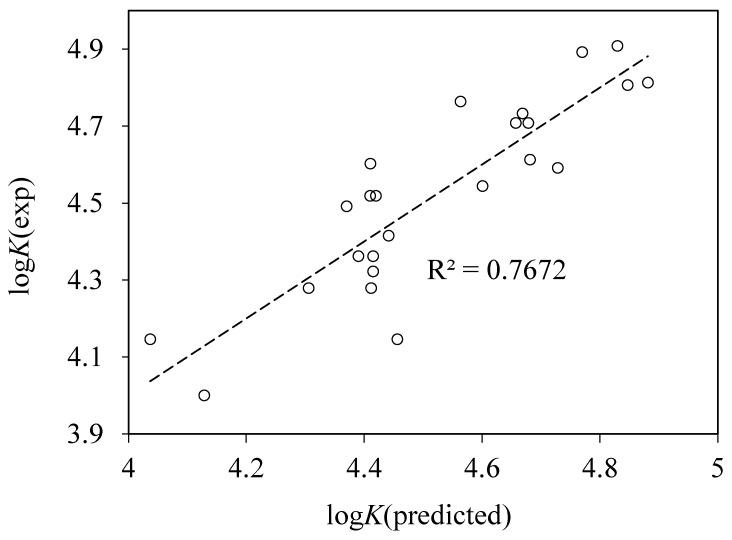
Plot of the experimental vs. predicted logarithms of binding constants log*K* obtained using the best two-parameter model (Equation (10)) with parameters from eDragon descriptor set.

With the second set of descriptors, we have also performed an exhaustive search of two-parameter regressions (671,061 combinations). The first 36 correlations with the largest *r*^2^ values included either HATS7v or HATS7u descriptors. The best correlation (Figure 8) is given by:

log*K* = −10,274 HATS7v + 0.26DP13 + 4.415. RMS = 0.117, *n* = 24, *r*^2^ = 0.767 (10)

The best of three-parameter models with HATS7v as one of the descriptors is given by equation:

log*K* = −9.095HATS7v + 0.202Mor06e + 0.307DP14 + 4.781. RMS = 0.1, *n* = 24, *r*^2^ = 0.829 (11)

## 5. Conclusions

Binding constants of substituted benzoic acids to BSA are sensitive to the nature and position of the substituents. The electron-density distribution in aromatic ring exerts crucial influence on the affinity of the acid anions, which is confirmed by correlation of the binding constants with the Hammett constants of substituents in m- and p-positions. While the Hammett constants or any other single ligand parameter cannot be used to describe binding of o-isomers, QSAR models with even as few as two descriptors are quite successful, and a three-descriptor model allowed to achieve the *r*^2^ value of 0.85 for 24 different acids. Further accumulation of the data on albumin binding constants of more chemically diverse sets of substances are necessary to develop general QSAR models, which can be useful for virtual screening of the affinity of drug candidates to blood plasma. However, only the constant values obtained using a uniform experimental methodology and data analysis procedure can be used for the development of such models. Numerous results from the previously published studies of albumin binding are unreliable and often inconsistent with each other even for the same system due to the strong dependence on the measurement technique.

## Figures and Tables

**Table 1:** Measured values of the binding constants of substituted benzoic acids with bovine serum albumin (BSA) at 298 K.

Ligand	*K*∙10^−4^
benzoic acid	1.9 ± 0.1
2-hydroxybenzoic acid	4.0 ± 0.2
3-hydroxybenzoic acid	1.9 ± 0.1
4-hydroxybenzoic acid	1.0 ± 0.1
2,4-dihydroxybenzoic acid	3.1 ± 0.2
2-nitrobenzoic acid	2.1 ± 0.2
3-nitrobenzoic acid	7.8 ± 0.3
4-nitrobenzoic acid	8.1 ± 0.2
2,4-dinitrobenzoic acid	6.5 ± 0.2
2-aminobenzoic acid	2.3 ± 0.2
3-aminobenzoic acid	1.4 ± 0.2
2-fluorobenzoic acid	3.3 ± 0.2
3-chlorobenzoic acid	5.8 ± 0.3
4-chlorobenzoic acid	5.1 ± 0.1
2-bromobenzoic acid	2.3 ± 0.2
3-bromobenzoic acid	3.5 ± 0.3
4-bromobenzoic acid	5.4 ± 0.2
2-iodobenzoic acid	3.3 ± 0.3
3-iodobenzoic acid	5.1 ± 0.2
4-iodobenzoic acid	4.1 ± 0.3
2-cyanobenzoic acid	2.6 ± 0.1
3-cyanobenzoic acid	3.9 ± 0.2
4-cyanobenzoic acid	6.4 ± 0.2
4-methylbenzoic acid	1.4 ± 0.1

**Table 2:** Binding constants of the studied benzoic acids with BSA and human serum albumin (HSA) previously reported in literature (at 298 K if not otherwise mentioned). The letters in brackets indicate the method of measurement (CE—affinity capillary electrophoresis, SP—spectrophotometry, SF—spectrofluorimetry, UC—ultracentrifugation, ED—equilibrium dialysis, CS—capacitive sensing technique).

Acid	*K*·10^−4^	
	BSA	HSA
2-hydroxybenzoic acid	3.92 (CE) [22], 7.38 (SF) [22], 10.0 (SP, 310 K) [9], 1.95 (SF) [11]	5.99 (CE) [22], 1.89 (CS) [22], 190 (SF) [23], 1.95 (SF) [10]
3-hydroxybenzoic acid	5.01 (SP, 310 K) [9], 0.85(SF) [11]	0.34 (SF) [10]
4-hydroxybenzoic acid	0.79 (SP, 310 K) [9], <0.1 (SF) [11], 0.68 (UC, 288 K) [8]	<0.1 (SF) [10], 0.45 (UC, 288 K) [8]
2,4-dihydroxybenzoic acid	<0.1 (SF) [11]	0.16 (SF) [10]
4-nitrobenzoic acid	6.03 (UC, 288 K) [8], 3.09 (ED, 310 K) [24]	8.13 (UC, 288 K) [8]
2-aminobenzoic acid	0.63 (SP, 310 K) [9]	
3-aminobenzoic acid	0.32 (SP, 310 K) [9]	
3-chlorobenzoic acid	11.22 (ED, 310 K) [24]	
4-chlorobenzoic acid	14.13 (UC, 288 K) [8], 14.96 (ED, 310 K) [24]	14.8 (UC, 288 K) [8]
2-bromobenzoic acid	3.98 (UC, 310 K) [9]	
3-bromobenzoic acid	63.1 (UC, 310 K) [9]	
4-bromobenzoic acid	20.0 (UC, 310 K) [9], 20.42 (ED, 310 K) [24]	
4-cyanobenzoic acid	1.66 (UC, 288 K) [8]	2.75 (UC, 288 K) [8]
4-methylbenzoic acid	7.94 (SP, 310 K) [9], <0.1 (SF) [11], 6.76 (UC, 288 K) [8], 3.16 (ED, 310 K) [24]	0.35 (SF) [10], 3.63 (UC, 288 K) [8]
benzoic acid	5.01 (SP, 310 K) [9], 2.24 (UC, 288 K) [8], 1.15 (ED, 310 K) [24]	2.29 (UC, 288 K) [8]

## Supplementary Materials

The following are available online at https://www.mdpi.com/1424-8247/13/2/30/s1, Script: Numerical simulation of Stern-Volmer plot for titration of 4-nitrobenzoic acid.
